# Exploring Relationship Between Immunocompetence, HPA Axis Functioning and Performances of Preweaning Dairy Calves

**DOI:** 10.3390/ani14243708

**Published:** 2024-12-23

**Authors:** Alessio Cotticelli, Giovanna Bifulco, Matilde Giombolini, Isabella Pividori, Alfio Calanni Macchio, Gianluca Neglia, Mirco Corazzin, Alberto Prandi, Tanja Peric

**Affiliations:** 1 Department of Veterinary Medicine and Animal Production, Federico II University, 80137 Naples, Italy; alessio.cotticelli@unina.it (A.C.); giovanna.bifulco@unina.it (G.B.); alfio.calannimacchio@unina.it (A.C.M.); neglia@unina.it (G.N.); 2 Department of Agricultural, Environmental and Animal Science, University of Udine, 33100 Udine, Italy; matilde.giombolini@uniud.it (M.G.); mirco.corazzin@uniud.it (M.C.); alberto.prandi@uniud.it (A.P.); tanja.peric@uniud.it (T.P.)

**Keywords:** transfer of passive immunity, management practices, hair, allostatic load, resilience, welfare, growth performances

## Abstract

Cortisol and DHEA(S) are markers of allostatic load and resilience; as such, they may be added to the definition of performance measures alongside immunocompetence. We aimed at studying the potential carryover effects of management practices on growth performances and the interrelationship between the hypothalamic–pituitary–adrenal (HPA) axis, immunocompetence and performances during the preweaning period in dairy calves. This study consisted of first a retrospective cohort study and second a prospective study. Calves had their growth performances and disease occurrence recorded, and their hair steroids (T1 at 20.03 ± 0.39 and T2 at 50.83 ± 0.41 days from birth) and plasmatic and colostral immunoglobulin G (IgG) was measured by immunoassays. The occurrence of preweaning diseases and the duration of individual housing negatively correlated to body weight (BW) at 60 days and the hair cortisol-to-DHEA(S) ratio (HC/HDHEA(S)) (T2) was negatively correlated to the daily weight gain (DWG) (−0.36; *p* < 0.05), plasmatic IgG (plaIgG) and the apparent efficiency of absorption (AEA) (−0.41; *p* < 0.01). Our results confirmed that the occurrence of diseases during the preweaning period and individual housing duration are pivotal for calves’ growth. This prospective study highlighted a detrimental effect of allostatic load on immunocompetence and described a link between resilience and growth performances.

## Simple Summary

The aim of the present study was to investigate the potential carryover effects of management practices on growth performances and the interrelationship between the hypothalamic–pituitary–adrenal (HPA) axis, immunocompetence and performances in dairy calves. Our results highlighted that the occurrence of diseases during the preweaning period and the duration of individual housing were negatively related to body weight (BW) at 60 days of life; moreover, the hair cortisol-to-DHEA(S) ratio (HC/HDHEA(S)) (T2) was negatively correlated to daily weight gain (DWG) (−0.36; *p* < 0.05), plasmatic immunoglobulins G (plaIgG) and the apparent efficiency of absorption (AEA) (−0.41, *p* < 0.01). Hence, we confirmed that the occurrence of diseases during the preweaning period and individual housing duration are pivotal for calves’ growth. Also, we highlighted an effect of allostatic load on immunocompetence and a link between resilience and growth performances. It was concluded that the last trimester of pregnancy and the preweaning period are crucial to develop an appropriate HPA axis functioning that may influence calves’ future performances that impact on farms’ profitability.

## 1. Introduction

The performance measures currently available for dairy calves may not always be sufficient well-being indicators during certain critical times [1]. In particular, the first days of life and the first two weeks after birth are recognized as two critical postnatal windows [2,3]. During the perinatal period, dairy calves must face many potential stressors including but not limited to birth, the transition from intrauterine to extrauterine life, cow–calf separation, dehorning and commingling [4]. So, standard management practices must help calves develop a robust stress response coupled with immunocompetence during the critical window around calving [5].

Among the standard management practices, colostrum administration is of utmost importance. The colostrum is the first milk secretion from dams after calving [6] and its high immunoglobulin content, especially of IgG, represents the first source of immunity [7]. The absorption of antibodies in calves is promoted by the 3Q rule: the administration of good-quality (>50 mg/mL of IgG) colostrum must be quick (within 4 h from birth) and in an appropriate quantity (more than 4 L during the first 12 h of life) [8]. Calves that are resilient and immunocompetent are characterized by a well-functioning hypothalamic–pituitary–adrenal (HPA) axis [9], physiological functionality and coping behaviors in presence of multiple stressors [1]. Conversely, offspring from stressed dams have protracted alterations in their resilience asset and immunocompetence [10,11], as also confirmed in foals and mares by measuring hair cortisol and dehydroepiandrosterone–sulfate (DHEA-S) concentrations [12]. As a matter of fact, cortisol and DHEA(S) play a pivotal role in triggering the process of parturition in ruminant species and driving the transition to extrauterine life [13,14]. On the other hand, elevated cortisol concentrations due to the chronic activation of the HPA axis leads to reduced growth and productivity [15,16]. The measurement of the above-mentioned steroids in the hair matrix provides a retrospective and long-term description of the HPA axis’s activity [17,18], and recently, their ratio has been investigated for a more accurate assessment [19]. Several studies have been conducted on cortisol and DHEA(S) in dairy cattle under different conditions [20]; still, only recent research has focused on calves [18,19,20,21,22,23]. As markers of allostatic load and resilience, these two steroids may be incorporated into the definition of performance measures along with immunocompetence. As a matter of fact, traditional performances, such as body weight, average weight gain and feed intake limit the evaluation of housing and management, especially during the first weeks of life, when the growth rate is highly variable [1]. In addition, cortisol and DHEA(S) may help in developing a more comprehensive assessment of metabolic status and growth responses in calves [24]. Despite recent advances in the management and housing of dairy calves, still more research is needed regarding the interrelationship between immunocompetence and HPA axis, possibly elucidating the physiological and behavioral responses of calves to stressors and reduce the risk of morbidity and mortality [1].

Hence, two experiments were conducted: a retrospective cohort study aimed at studying the potential carryover effects of management practices on the growth performances of dairy calves; and a prospective study designed to investigate the calves’ HPA axis functioning in relation to (i) immunocompetence, (ii) the HPA axis activity of their dams, and (iii) growth performances during the preweaning period.

## 2. Materials and Methods

This study consisted of two experiments: the first was a retrospective cohort study and involved 914 calves born between January 2018 and January 2021. Afterwards, the second experiment consisted of a prospective study conducted between March and June 2021 and focused on 60 animals (30 calves and 30 dams). Both experiments were carried out at a commercial dairy farm located in Roveredo (UD, Friuli Venezia Giulia region, Italy), where approximately 1300 Holstein Friesians cattle are bred.

### 2.1. Ethics

This study obtained institutional approval from the Ethical Animal Care and Use Committee of the University of Naples Federico II (protocol no. PG/2021/0130478). All experimental procedures complied with the Italian legislation on animal care (Legislative Decree n.26 of 04/03/2014).

### 2.2. Experiment 1: Retrospective Cohort Study

The retrospective cohort study included a dataset of 914 Holstein Friesians calves born between January 2018 and January 2021. The calves were individually housed for the first 12.32 ± 0.15 days of life and fed manually twice per day with teat buckets with 7.22 ± 0.02 L/day (mean ± standard error (SE)) of milk replacer (Sprayfo royal 60, Trouw Nutrition, Amersfoort, The Netherlands) reconstituted at 140 g powder/L. Then, they were moved on to straw in group pens (10 individuals/20 m^2^) equipped with an automatic milk feeder (Urban Alma Pro, Urban GmbH & Co. KG, Wüsting, Germany) with an individual RFID (radio frequency identification) system and supplemented by pelleted starter feed (Fly Start, Cortal Extrasoy S.p.A., Cittadella, PD, Italy) ad libitum until weaning (day 68). The weaning process consisted of a gradual reduction in milk replacer over 3 weeks. The birth (day, season and year), individual housing duration (days), colostrum and milk (volume, L) fed throughout the weaning period, body weights (BW (kg) at birth and at 60 days of life) and occurrence of preweaning diseases (respiratory syndromes and perinatal diarrhea) were recorded. The daily weight gain (DWG) was calculated by subtracting the birth weight from the weight at 60 days and dividing by the age (days). The diseases were diagnosed by farm professionals trained by herd veterinarians and were based on a health scoring system [25]. Namely, a diarrhea score encompassing fecal scores of 2 as loose or 3 as watery and a respiratory score of 5 or above were used to differentiate animals affected by diarrhea or bovine respiratory disease (BRD) from unaffected ones.

### 2.3. Experiment 2: Prospective Study

The prospective study was conducted between March and June 2021 in the above-described commercial dairy farm. Thirty Holstein Friesians calves and their dams (n = 30; age 2.76 ± 0.19 years; parity 1.87 ± 0.17; mean ± SE) were used. The cattle were kept in boxes with concrete floor during the close-up (10 days before the expected delivery) and fed a diet that met the National Research Council (NRC) requirements [26]. Every cow enrolled in the experiment was healthy and had no signs of mastitis, metabolic disorders or previous history of dystocia. The somatic cell count of the previous lactation and the BRIX score (27 or above) were also considered. The colostrum was obtained at the first milking after calving from each dam and administered via a bottle to their calves (at least 3 L (10–12% of body weight according to Godden et al. [27])). Only healthy calves born to eutocic dams were included in the study. The calves were individually housed for the first 10.50 ± 0.78 days of life and fed manually twice per day with teat buckets with 8.22 ± 0.17 L/day (mean ± SE) of milk replacer (Sprayfo royal 60, Trouw Nutrition, Amersfoort, The Netherlands). Then, they were moved on to straw in group pens equipped with an automatic milk feeder and supplemented by pelleted starter feed (Fly Start, Cortal Extrasoy S.p.A., Cittadella, PD, Italy) ad libitum until weaning (day 68). The aforementioned weaning process was followed.

The birth date and time (hour), colostrum and milk fed throughout the preweaning period (volume, L) and feeding time were recorded. In addition, the calves had their body weight (BW, kg) recorded at birth and at 60 days of life using an electronic scale; the DWG was calculated accordingly.

#### 2.3.1. Environmental Temperature, Relative Humidity and THI

Climatic data were collected from a weather station located 8 km from the farm (provided by ARPA FVG, Agenzia Regionale per la Protezione dell’Ambiente del Friuli Venezia Giulia, Italy) and used for the temperature humidity index (THI) calculation [28] (1):

THI = (1.8 × T + 32) − (0.55 − 0.55 × RH) × [(1.8 × T + 32) − 58](1)

where T is the environmental temperature expressed in degrees Celsius.

The expression (1.8 × AT + 32) represents the conversion of temperature data into degrees Fahrenheit.

RH is the relative humidity as a fraction of a unit.

The environmental temperature and relative humidity were recorded every hour, and the hourly THI values during the day were used for the daily average THI calculation.

#### 2.3.2. Colostrum and Blood Samplings and Immunoglobulin Assay

The colostrum was individually collected from the dams at the first milking after calving in sterile Falcon tubes (Falcon^®^ 50 mL, Corning Science, Reynosa, Mexico) and stored at −20 °C. Prior to administration, the colostrum quality was measured using a refractometer (Sper Scientific, Scottsdale, AZ, USA) and expressed as a Brix value. The calves underwent blood sampling within 72 h from birth according to the IgG dynamic described by Osaka et al. [29]. Blood (10 mL) was collected via jugular venipuncture in vacutainer tubes (lithium heparin) and immediately centrifuged at 1500× *g* for 15 min. The plasma was aliquoted into Eppendorf tubes (1 mL) and stored at −20 °C until analyses were performed.

Colostral (coIgG) and plasmatic IgG (plaIgG) were assayed using the IgG ELISA kit (Cat. no. E11-118; Lot no. E11-118-200904; Bethyl Laboratories Inc., Montgomery, TX, USA) validated for bovine species. The assay range declared by the manufacturer is 0.69–500 ng/mL and the sensitivity is 0.69 ng/mL. Colostrum and blood were serially diluted following the scheme proposed by the manufacturer; the final dilutions were 1:500,000 for the colostrum and 1:250,000 for the plasma. The assay was carried out following the manufacturer’s instructions. Absorbance was read using a multimode plate reader (Ensight, Perkin-Elmer Life Science, Boston, MA, USA) at a wavelength of 450 nm.

The concentrations of coIgG and plaIgG (ng/mL), calf weight (kg) and colostrum volume (L) were used to calculate the apparent efficiency of absorption (AEA) of each calf using the following equation [30] (2):

AEA = (((plaIgG (g/L) × BW (kg) × 0.7(estimated % blood volume)))/coIgG (g/L) × colostrum administered (L)) × 100(2)

#### 2.3.3. Hair Sampling and Steroids Assay

Hair was collected from the scapular region by shaving close to the skin using an electric clipper and kept in paper envelopes in the dark at room temperature until the analysis. Samples were taken at 20.03 ± 0.39 (T1) and 50.83 ± 0.41 (T2) days from birth for calves and at 2.71 ± 0.21 (T1) and 19.65 ± 0.40 (T2) days from parturition for cattle, according to the shave–reshave technique [31].

The hair samples were washed and extracted before the steroid assay as described by Peric et al. [32]. Briefly, the hair samples were washed in ultra-pure water (5 mL) and then in isopropanol (5 mL, Sigma-Aldrich, St. Louis, MO, USA). Then, approximately 60 mg of hair was extracted in glass vials with methanol (3 mL, Sigma-Aldrich, St. Louis, MO, USA) and incubated for 16 h at 37 °C. Vials were evaporated to dryness under an airstream suction hood; the residues in the tubes were dissolved with 0.05 M phosphate-buffered saline (PBS) at a pH of 7.5.

The hair cortisol (HC) was measured using a solid-phase microtiter radioimmunoassay (RIA) as described by Peric et al. [32]. The rabbit anti-cortisol antibody (Analitical Antibodies, Bologna, Italy) showed the following cross-reactivities: cortisol, 100%; cortisone, 4.3%; corticosterone, 2.8%; 11-deoxycorticosterone, 0.7%; 17-hydroxyprogesterone, 0.6%; dexamethasone, 0.1%; progesterone, <0.01%; 17-hydroxypregnenolone, <0.01%; DHEA-S, <0.01%; androsterone sulfate, <0.01%; and pregnenolone, <0.01%. For the cortisol, the intra- and inter-assay coefficients of variation (CV) were 3.7% and 10.1%, respectively. The sensitivity of the assay, calculated as the interpolated dose of the response to a concentration of zero minus the statistical error, was 24.6 pg/mL.

The hair DHEA(S) (HDHEA(S)) concentrations were measured using a solid-phase microtiter RIA. In brief, a 96-well microtiter plate (OptiPlate; PerkinElmer Life Sciences Inc., Milan, Italy) was coated with goat anti-rabbit g-globulin serum diluted at 1:1000 in 0.15 mM of sodium acetate buffer (pH of 9) and incubated overnight at 4 °C. The plate was then washed twice with RIA buffer (pH of 7.54) and incubated overnight at 4 °C with 200 mL of the antibody serum diluted at 1:40.000. The cross-reactivities of the antibody (Analitical Antibodies, Bologna, Italy) with other steroids were as follows: DHEA-S, 100%; DHEA, 100%; DHEA 3-glucuronide, 15%; androstenedione, 5.9%; pregnenolone, 0.3%; epiandrosterone 3-glucuronide, 2.7%; androsterone sulfate, 2.9%; cortisone, <0.001%; cholesterol, 0.00001%; and cholesterol oleate, 0.00001%. After washing the plate with RIA buffer, standards (5–200 pg/well), a quality control extract, test extracts, and a tracer (DHEAS [1,2,6,7-3H (N)]; Perkin-Elmer Life Sciences Inc., Milan, Italy; specific activity: 70.5 Ci/mmol; 20 pg/well) were added, and the plate was incubated overnight at 4 °C. Bound hormones were separated from free hormones by decanting and washing the wells in RIA buffer. After the addition of 200 mL of a scintillation cocktail, the plate was counted using a ß-counter (Top-Count, Perkin-Elmer Life Sciences Inc., Milan, Italy). The intra- and inter-assay CVs were 3.2% and 11.8%, respectively. The sensitivity of the assay, calculated as the interpolated dose of the response to a concentration of zero minus the statistical error, was 10.8 pg/mL. To determine the parallelism between the standard curve and endogenous DHEA(S), in hair containing high concentrations of endogenous hormones, the samples were serially diluted in 0.05 M PBS at a pH of 7.5. The relationship between the hair DHEA(S) concentrations and the standard curve determined through linear regression was linear; the correlation coefficient (r) was 0.99 and the model was given by the equation y = 0.95x + 4.80. A recovery test was conducted to evaluate the system’s response to an increasing amount of DHEA(S) standard added to a hair extract with low DHEA(S). The percentage of recovery was determined as follows: [(measured DHEA(S) in spiked sample)/(measured DHEA(S) in non-spiked sample + DHEA(S) added) × 100]. In the recovery test, the recovery rate was 96 ± 6% (mean ± SD).

### 2.4. Statistical Analysis

Statistical analyses were conducted using SPSS (29.0.1.1) for Windows 10 (SPSS Inc., Chicago, IL, USA). The compliance of the data with a normal distribution was assessed by the Shapiro–Wilk test. The data recorded during the first experiment were studied by multivariate analysis of variance (general linear model) with the season and year as fixed factors; the birthweight (kg), individual housing duration (days), colostrum and milk fed (volume, L) throughout the preweaning period, and daily weight gains (1–60 days) were the dependent variables. The interaction between the year and season was also considered. The relationships between the occurrence of preweaning diseases (as a binary variable), weight and individual housing duration were studied by logistic regressions. The relationships between the variables included in the logistic regression were analyzed by using contingency tables. The Mann–Whitney U test was used to compare the steroid concentrations measured within the second experiment, and Spearman’s rank correlation was used to study the relationships between colostral and plasmatic IgG, the apparent efficiency of absorption, hair steroid concentrations in the calves, and growth performances. Unless otherwise stated, results are expressed as the mean ± SE. A statistically significant difference was accepted at *p* < 0.05, and tendencies were discussed at 0.05 < *p* ≤ 0.10.

## 3. Results

Throughout the retrospective study, the calves received 3.51 ± 0.02 L of colostrum (mean/head ± SE) within the first 3 h after birth and 617.45 ± 2.00 L of milk replacer (mean/head ± SE) until weaning.

A descriptive statistic of the data recorded during the retrospective cohort study is reported in Table 1.

The multivariate analysis of variance performed on the data recorded in the retrospective study showed that the BW at 60 days was highly influenced by the year, individual housing duration, occurrence of preweaning diseases (*p* > 0.01) and season (*p* = 0.06). The Phi and Cramer’s V were, respectively, 0.167 and 0.096, revealing no significant relationship between the individual housing duration and occurrence of preweaning disease. The occurrence of a preweaning disease and the duration of individual housing (ranging between 5 and 27 days) were negatively related to the distribution of the BW at 60 days, with calves spending an additional day in an individual cage (OR = 1.05; *p* = 0.02) or contracting a disease (OR = 2.78; *p* < 0.01) during the preweaning period being 5% and 178% more likely to show a BW at 60 days under the fifth percentile.

The THI values during the prospective study ranged between 40.41 ± 3.68 in January and 71.90 ± 3.25 in June. The calves received 3.40 ± 0.08 L (mean ± SE) of colostrum (27.97 ± 0.34%, mean Brix score ± SE) within the first 3 h of life and 639.63 ± 6.09 L of milk replacer (mean/head ± SE) up to weaning. As regards the performances, the calves showed a weight of 37.58 ± 0.21 and 91.43 ± 1.35 kg at birth and day 60, respectively; accordingly, the DWG ranged between 0.66 and 1.17 kg/day (0.91 ± 0.02 kg/day (mean ± SE)).

The IgG ranged between 1.31 mg/mL and 72.30 mg/mL in the plasma and between 29.30 mg/mL and 268.70 mg/mL in the colostrum. The apparent efficiency of absorption showed a minimum of 0.67% and a maximum of 60.37%. Moreover, 56.67% of the calves were characterized by an AEA between 15.67 and 45.67%.

The hair steroid concentrations of the calves are reported in Table 2: the HC, HDHEA(S) and HC/HDHEA(S) ratio differed significantly between the sampling times (*p* < 0.01 and *p* < 0.05, respectively).

**Figure 1:**
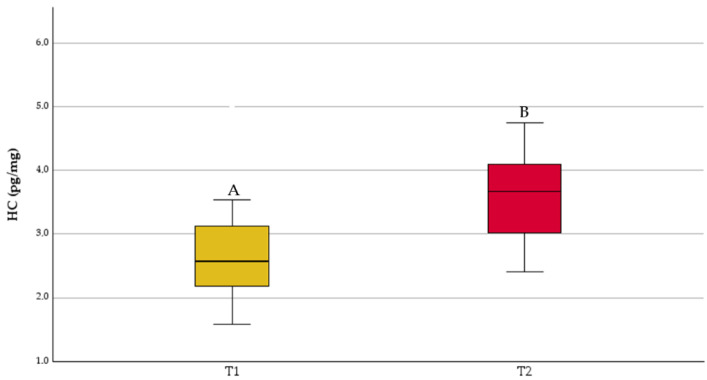
Hair cortisol (HC) of dams at parturition (growth hair; T1 = 2.71 ± 0.21 days from parturition) and 15 days later (regrowth hair; T2 = 19.65 ± 0.40 days from parturition). Different letters indicate significant differences (A, B: *p* < 0.01).

The HC of the dams is shown in Figure 1; higher concentrations were measured at T2 (3.78 ± 0.24 pg/mg) compared to T1 (2.76 ± 0.17 pg/mg; *p* < 0.01).

Table 3 includes the Spearman correlation coefficients between the calves’ hair steroid concentrations, IgG, AEA and growth performances. The DWG was correlated both to the HDHEA(S) (T1) (0.51; *p* < 0.01) and HC/HDHEA(S) ratio (T1) (−0.52; *p* < 0.01). The HC/HDHEA(S) ratio (T2) was negatively correlated to the DWG (−0.36; *p* < 0.05), IgG (pla) and AEA (−0.41; *p* < 0.01).

Eventually, a negative correlation emerged between the AEA and HC (−0.34; *p* < 0.07) at T2. The HC and HDHEA(S) were positively correlated (0.33 (*p* < 0.08) at T1 and 0.74 (*p* < 0.0001) at T2).

## 4. Discussion

The present studies aimed at studying the potential carryover effects of management practices and health scores on the growth performances of dairy calves (retrospective study) and investigating the HPA axis of calves in relation to immunocompetence, the HPA axis activity of dams and growth performances throughout the preweaning period (prospective study). Throughout the retrospective study, we focused on two health issues with higher incidence during the first weeks of life, namely diarrhea and respiratory disease.

In previous research [33], pneumonia has been associated with a reduction in the average daily weight gain; similarly, a failure in passive immune transfer reduced the average daily weight gain during the first month of life in dairy calves. Moreover, the same authors reported positive effects of the optimal transfer of passive immunity on calves’ preweaning health and postweaning reproductive efficiency. On the other hand, neither disease nor the failure of passive immune transfer were reported to affect average daily weight gain during the second month [34]. Conversely, Donovan and colleagues [35] showed that the season of birth and the occurrence of diarrhea, septicemia and respiratory disease at birth significantly decreased heifers’ growth. In addition, according to Place et al. [36], housing and the season significantly influence average daily weight gain from birth to 4 months of age. More recently, Heinrichs et al. [37] found that the age at the first calving and body weight at calving were affected by nutrition, housing factors and the management of the calf during the first 16 weeks of life. Lastly, Ettema and Santos [38] showed that the age at the first calving can even affect first-lactation milk production, with both early and late calving ages being characterized by less production.

Based on this rationale, our retrospective study was conducted to investigate the components of the heifer-raising system that may have affected the growth performances of dairy calves (subjected to appropriate colostrum management) in the selected farm over the previous three years. Our results confirmed that the occurrence of diseases such as respiratory syndromes and diarrhea (or the concurrence of the two) during the preweaning period was highly correlated to the body weight of the dairy calves at 60 days of life. Furthermore, the duration of the period spent in individual housing seemed pivotal for the growth of the calves, since it was related to the body weight at 60 days. It is generally known that the standard practice in the dairy industry is to separate the calf and dam immediately after birth and raise calves in individual pens during the milk-feeding period. In the present day, however, both consumers’ and welfare concerns are rising as regards maternal separation and the social isolation of calves. Costa et al. [39] summarized the available scientific knowledge on the effects of social isolation on calves and reported behavioral and developmental harm associated with individual housing; conversely, intakes and weight gains were improved with social housing. The evidence of the present retrospective study integrated existing knowledge about the negative relationship between social isolation, preweaning pathologies and growth performances and connected with the up-to-date EFSA’s Scientific Opinion on the Welfare of Calves [40] that recommends prolonged cow–calf contact and small groups of 2–7 animals during the first week of life and stable groups thereafter.

Then, the prospective study was designed to investigate the interrelationship between the HPA axis functioning, immunocompetence and growth performances during the sensitive preweaning period.

The hypothesis was that measurements of allostatic load and immunocompetence may be incorporated into performance measures to provide a more comprehensive assessment of calves’ phenotype. As a matter of fact, the HPA axis and immune system develop together, leading to an immunocompetent and resilient calf; nevertheless, the measurements of stress, resilience and immunocompetence are still not included in the definition of performance measures [1]. In addition, the whole preweaning period represents a critical biological window that may affect the performance and overall well-being of calves for their entire life [1].

Since Uetake et al. [41] suggested that calves born by natural delivery could better cope with subsequent stress after birth compared to calves born to cows with severe and/or prolonged stress (i.e., difficult delivery), only healthy calves born without dystocia were included in this study and the allostatic load of dams during peripartum was investigated to avoid any potential bias and exclude the influence of maternal stress.

Moreover, the THI was a factor considered in the experimental design since it influences the weight gain and allostatic load of calves [42] and colostrum quality [43]. Roland et al. [44] reported that any deviation from the thermoneutral zone for a calf causes different degrees of thermal stress, although limited information is available for calves and heifers [42]. Throughout the prospective study, the THI did not affect the parameters investigated, probably because it remained below the heat stress threshold of 72 described for dairy cattle by Bernabucci et al. [28]. The daily weight gain of the calves was recorded throughout the prospective study since it is a key performance indicator associated with age and weight at the first calving, lifetime productivity [45], financial and carbon efficiency, rearing costs and first-lactation milk yields [46]. In the context of the second experiment, the growth performances recorded were considered excellent according to the average daily gain thresholds described by Shivley et al. [47] for pre-weaned Holstein calves.

Hence, the hypothesis of the prospective study was that a less efficient absorption and a subsequent lowered plasmatic concentration of immunoglobulins could lead to an impaired anabolic/catabolic balance and long-term effects on allostasis; vice versa, an increment in the cortisol/DHEA(S) ratio could be detrimental for the immune system.

In the present study, the plaIgG concentrations of the calves were comparable to those reported by Zwierzchowski et al. [48] and below the prevalence of the failure of the transfer of passive immunity (19.2% in dairy heifers according to Beam et al. [49]) with only three out of thirty calves showing concentrations <10 mg/mL. Similarly, the coIgG concentrations’ range was comparable to the one reported by Costa et al. [8]. Consequently, the percentage of calves characterized by an AEA between 15.67 and 45.67% was in agreement with Halleran et al. [30] who reported similar results with 69% of calves with an AEA falling between 21 and 40%. Therefore, it can be concluded that appropriate colostrum management was conducted throughout the second experiment and it did not negatively affect the performances of the calves. So, hair steroid measurements were performed to address the hypothesis regarding the HPA axis reactivity during the sensitive preweaning window.

The trichological matrix was the matrix of choice, since it integrates baseline concentrations and short-term peaks of steroids over longer periods, up to several months [20]. Within the prospective study, the first hair sample of the calves pictured the last third of intrauterine life and the perinatal period (T1), while the second described the subsequent 30 days of extrauterine life (T2, regrown hair); the last third of pregnancy (T1) and the early post-partum period (T2, regrown hair) were, respectively, characterized by the first and second hair samples of the dams [31].

Considering the time required by the hair section to reach the skin layer, the hair of the calves was sampled for the first time 15 days after the birth to picture the first days of extrauterine life too [50]. On the other hand, the second hair sampling was performed within 15 days from the first in the dams to differentiate prepartum from postpartum samples. Also, the regrown hair after two weeks allowed us to have proper quantity of hair for the immunoassay, with the growth rate of hair in Holstein cows being 0.30 mm/day at the shoulder [51].

To the best of the authors’ knowledge, this is the first study assessing hair steroid concentrations during the perinatal and preweaning period in dairy calves. The hair steroid concentrations were higher at T1 in the calves (*p* < 0.01). The route by which parturition is initiated in cows is triggered by fetal ACTH that stimulates the adrenal gland to increase cortisol secretion. It is transferred through the placenta, as reported by Uetake et al. [41], with positive correlations between the plasma cortisol concentrations of dams and their calves. In hair, neonatal glucocorticoids are influenced by the third-trimester increase in HPA axis activity [52]. The cortisol concentrations measured at T1 in the present study were consistent with the sharp decrement in hair cortisol observed after birth in humans by Hollanders et al. [53], with the highest hair cortisol at birth in beef cattle reported by Probo et al. [18] and with the effect of age on the hair cortisol concentrations of dairy cattle found by González-de-la-Vara et al. [54]. Accordingly, the hair at birth and the first days of extrauterine life showed the highest concentrations of DHEA(S). Thereafter, a significant (*p* < 0.01) reduction was observed at T2; a comparable trend was reported in beef calves by Probo et al. [18]. In humans, a peak in circulating fetal concentrations of DHEA(S) has been described in the pregnancy term, succeeded by a rapid decline after birth; hence, DHEA(S) physiologically follows cortisol’s trend [55]. Since DHEA(S) has anti-glucocorticoid properties, it acts as an “anti-stress” steroid minimizing negative glucocorticoid effects [56] and it is released concurrently with cortisol by adrenal glands in response to ACTH as a result of HPA axis activity to perform its neuroprotective role [57,58]. The HDHEA(S) recorded at T1 suggests a high resilience displayed during the perinatal period that is characterized by radical physiological changes and a significant allostatic load to deal with for the newborns [18,19,20,21,22,23,24,25,26,27,28,29,30,31,32,33,34,35,36,37,38,39,40,41,42,43,44,45,46,47,48,49,50,51,52,53,54,55,56,57,58,59,60]. It must be underlined that postnatal physiological dynamics in human species are completely different compared to those of calves; therefore, the different perinatal DHEA(S) pattern must consider the prolonged exposure to potential stressors with which calves must cope. It is also in agreement with Probo et al. [18] who observed a higher HDHEA-S until six weeks after birth, suggesting that these steroids are triggered by events occurring during the challenging first weeks of life in bovine species. Accordingly, the cortisol/DHEA(S) ratio was higher during the perinatal period (T1; *p* < 0.05), when the calf must face a number of stressors, testing its coping ability [61]. The ratio describes the HPA axis activity in a single piece of information, and it has been used to indicate impaired fetal development in pregnant mares [62], as a biomarker of inflammatory foot lesions in bovine species [63] and to explore the influence of seasonality on the HPA axis in stallions [64]. Eventually, the strong positive correlation between the steroid concentrations at T2 confirmed the tight relationship between the two hormones within the HPA axis functioning, as already reported by Lanci et al. [62].

The hair cortisol concentrations of the dams were within the postpartum physiological range of the species [65], allowing us to exclude the detrimental influence of maternal stress on the newborns. The second hair sampling was performed to monitor the postpartum period of the dams; as expected, the early postpartum period (T2) was characterized by a higher allostatic load compared to the last third of pregnancy (T1), with the calving and the weaning processes being associated stress-inducing events [66].

The steroid biomarkers of HPA axis functioning have been previously linked to immune status; in particular, an altered HC/HDHEA(S) ratio has been connected to the age-related loss of immunity in humans [67,68,69] and DHEA to the preservation of immune function in thermally injured mice [70].

Investigating the relationship between the steroid biomarkers of the HPA axis and the transfer of passive immunity, the prospective study highlighted that the HC and HC/HDHEA(S) ratio of the calves at T2 were negatively correlated to the AEA and plaIgG. It is likely that allostatic load exerts a detrimental effect on immune status in bovine species, as already reported in human and mice [67,68,69,70].

The relationship between steroids and performances was also investigated to identify the potential carryover effect of HPA axis functioning on the growth performances of the dairy calves. The influence of DHEA(S) on weight gain has never been studied in animals so far. Nevertheless, two recent studies in humans showed a correlation between high serum concentrations of DHEA(S) and weight gain [71,72]. In our study, the HDHEA(S) at T1 was positively correlated to the DWG (*p* < 0.01). It seems likely that adequate resilience during the critical phases of the last third of intrauterine life and the perinatal period related to the later performances of the calves. Also, significant negative correlations emerged between the HC/HDHEA(S) ratio (for both sampling times) and the DWG. So, our results seem to suggest that the link between resilience and performance previously reported in other species may apply to bovine species too. In addition, the negative correlation with the performances of the calves seemed to confirm that the cortisol-to-DHEA(S) ratio could be more informative than either hormone alone [73]. Modern dairy farming is pushing towards sustainability and efficiency, so more and more advanced predictive methods are being developed to predict reproductive functionality and health and, consequently, to mitigate environmental impact [74,75]. Based on the results herein reported, the ratio may represent a useful tool to monitor the growth performance of calves; hence, more research is encouraged to investigate the cortisol-to-DHEA(S) ratio and performances outcomes in dairy calves. As potential limitations of the present study, our data originated from a single farm, which may limit this study’s external repeatability. Also, this was an observational study and it was not possible to establish cause–effect relationships. Finally, the number of calves included in the second experiment may not be representative of larger populations.

## 5. Conclusions

Our study confirmed how social isolation and preweaning diseases are negatively related to calves’ well-being and performances. Moreover, we verified that the last trimester of pregnancy and the perinatal period are essential to developing appropriate HPA axis functioning, which may influence calves’ future performances which are pivotal for farms’ profitability. More research is encouraged to elucidate the interrelationship between immunocompetence, the HPA axis response and performance outcomes, potentially including the contribution of genetic variance and robustness in such phenotypic measures.

## Figures and Tables

**Table 1:** The frequency distribution of the calves enrolled in the retrospective study across the different variables considered for building the statistical models. The preweaning diseases are the total syndromes diagnosed during the retrospective study (n = 216), split into respiratory (n = 173) and diarrhea (n = 43); concomitant (n = 6) indicates calves showing both syndromes simultaneously.

Variable	n	Frequency (%)
2018	269	29.43
2019	307	33.59
2020	309	33.81
2021 (until February)	29	3.17
Autumn	264	28.88
Summer	193	21.12
Winter	295	32.28
Spring	162	17.72
Preweaning diseases (total animals affected either by diarrhea or respiratory disease)	216	23.63
Diarrhea	43	4.70
Respiratory (Bovine Respiratory Disease)	173	18.93
Concomitant	6	2.78
Total	914	

**Table 2:** Hair cortisol (HC) and DHEA(S) (HDHEA(S)) concentrations and cortisol/DHEA(S) ratio (HC/HDHEA(S) ratio) measured during prospective study. Hair of calves was sampled at 20.03 ± 0.39 (growth, T1) and 50.83 ± 0.41 (regrowth, T2) days from birth.

Hair Steroids	T1	T2
HC (pg/mg)	15.49 ± 0.74 ^A^	11.14 ± 1.03 ^B^
HDHEA(S) (pg/mg)	167.59 ± 7.08 ^A^	137.00 ± 14.07 ^B^
HC/HDHEA(S) ratio	9.61 ± 0.53 ^a^	7.98 ± 0.48 ^b^

Different letters in the same row indicate significant differences (^A,B^ *p* < 0.01; ^a,b^ *p* < 0.05).

**Table 3:** Correlations between the hair cortisol (HC), hair DHEA(S) (HDHEA(S)) and cortisol-to-DHEA(S) ratio (HC/HDHEA(S) ratio) of the calves at T1 and T2; the performances (birth weight, body weight (day 60) and daily weight gain (DWG days 1–60)), plasmatic and colostral IgG (plaIgG and coIgG, respectively) and apparent efficiency of absorption (AEA) were recorded throughout the prospective study.

	HC (T1)	HDHEA(S) (T1)	HC/HDHEA(S) Ratio (T1)	HC (T2)	HDHEA(S) (T2)	HC/HDHEA(S) Ratio (T2)	Birth Weight	Body Weight (Day 60)	DWG (Days 1–60)	plaIgG	coIgG	AEA
HC (T1)	1.00	0.33	0.54	0.41	0.30	0.07	−0.19	−0.10	−0.07	−0.05	−0.11	0.03
*p*		0.08	0.00	0.03	0.10	0.69	0.33	0.60	0.70	0.77	0.57	0.86
HDHEA(S) (T1)	0.33	1.00	−0.54	0.19	0.32	−0.29	−0.06	0.47	0.51	0.10	0.20	0.03
*p*	0.08		0.00	0.31	0.08	0.12	0.74	0.01	0.00	0.58	0.29	0.88
HC/HDHEA(S) ratio (T1)	0.54	−0.54	1.00	0.14	−0.12	0.37	−0.04	−0.49	−0.52	−0.12	−0.25	0.03
*p*	0.00	0.00		0.46	0.54	0.04	0.85	0.01	0.00	0.53	0.18	0.88
HC (T2)	0.41	0.19	0.14	1.00	0.74	0.32	0.15	0.11	0.10	−0.35	0.16	−0.34
*p*	0.03	0.31	0.46		<0.001	0.09	0.42	0.56	0.60	0.06	0.40	0.07
HDHEA(S) (T2)	0.30	0.32	−0.12	0.74	1.00	−0.27	0.16	0.30	0.30	−0.06	0.16	−0.04
*p*	0.10	0.08	0.54	<0.001		0.15	0.39	0.11	0.10	0.77	0.40	0.81
HC/HDHEA(S) ratio (T2)	0.07	−0.29	0.37	0.32	−0.27	1.00	0.08	−0.31	−0.36	−0.41	0.02	−0.41
*p*	0.69	0.12	0.04	0.09	0.15		0.66	0.10	0.05	0.02	0.93	0.02
Birth weight	−0.19	−0.06	−0.04	0.15	0.16	0.08	1.00	0.47	0.32	−0.04	−0.24	0.18
*p*	0.33	0.74	0.85	0.42	0.39	0.66		0.01	0.08	0.84	0.20	0.35
BW (day 60)	−0.10	0.47	−0.49	0.11	0.30	−0.31	0.47	1.00	0.98	0.02	0.06	0.04
*p*	0.60	0.01	0.01	0.56	0.11	0.10	0.01		<0.001	0.94	0.75	0.81
DWG (days 1–60)	−0.07	0.51	−0.52	0.10	0.30	−0.36	0.32	0.98	1.00	0.05	0.12	0.01
*p*	0.70	0.00	0.00	0.60	0.10	0.05	0.08	<0.001		0.80	0.54	0.94
plaIgG	−0.05	0.10	−0.12	−0.35	−0.06	−0.41	−0.04	0.02	0.05	1.00	0.34	0.68
*p*	0.77	0.58	0.53	0.06	0.77	0.02	0.84	0.94	0.80		0.06	<0.001
coIgG	−0.11	0.20	−0.25	0.16	0.16	0.02	−0.24	0.06	0.12	0.34	1.00	−0.33
*p*	0.57	0.29	0.18	0.40	0.40	0.93	0.20	0.75	0.54	0.06		0.07
AEA	0.03	0.03	0.03	−0.34	−0.04	−0.41	0.18	0.04	0.01	0.68	−0.33	1.00
*p*	0.86	0.88	0.88	0.07	0.81	0.02	0.35	0.81	0.94	<0.001	0.07	

## Data Availability

The data presented in this study are available on reasonable request from the corresponding author.
